# Superpixel image segmentation of VISTA expression in colorectal cancer and its relationship to the tumoral microenvironment

**DOI:** 10.1038/s41598-021-96417-1

**Published:** 2021-08-31

**Authors:** Dongling Wu, Sean Hacking, Taisia Vitkovski, Mansoor Nasim

**Affiliations:** Department of Pathology and Laboratory Medicine, Donald and Barbara Zucker School of Medicine at Hofstra/Northwell, Hempstead, NY USA

**Keywords:** Cancer, Immunology, Gastroenterology, Oncology, Pathogenesis, Risk factors

## Abstract

Colorectal cancer (CRC) is the third most common cause of cancer related death in the United States (Jasperson et al. in Gastroenterology 138:2044–2058, 10.1053/j.gastro.2010.01.054, 2010). Many studies have explored prognostic factors in CRC. Today, much focus has been placed on the tumor microenvironment, including different immune cells and the extracellular matrix (ECM). The present study aims to evaluate the role of V-domain immunoglobulin suppressor of T cell activation (VISTA). We utilized QuPath for whole slides image analysis, performing superpixel image segmentation (SIS) on a 226 patient-cohort. High VISTA expression correlated with better disease-free survival (DFS), high tumor infiltrative lymphocyte, microsatellite instability, BRAF mutational status as well as lower tumor stage. High VISTA expression was also associated with mature stromal differentiation (SD). When cohorts were separated based on SD and MMR, only patients with immature SD and microsatellite stability were found to correlate VISTA expression with DFS. Considering raised VISTA expression is associated with improved survival, TILs, mature SD, and MMR in CRC; careful, well-designed clinical trials should be pursued which incorporate the underlying tumoral microenvironment.

## Introduction

As one of the major cancers in United States, most colorectal cancers (CRC) occur sporadically, while others can be inherited with deficient mismatch repair (MMR)^1^. Patients with microsatellite instability (MSI) have been shown to benefit from program death-ligand 1 (PD-L1) therapy, with Nivolumab being shown to have clinical response in the checkmate 142 clinical trial^2^. However, the rarity of metastatic MSI-high CRC^3^ has limited the widespread use of immune checkpoint blockade in clinical practice.

The world of immunotherapy is not limited to the PD-L1 axis, and we are working to further understand the complex ecosystem of immune escape in the tumoral microenvironment. One such marker is V-domain Ig suppressor of T cell activation (VISTA). Unlike PD-L1, VISTA is mainly expressed in stromal hematopoietic and myeloid cells; as well as naïve CD4+ and Foxp3+ regulatory T-cells^4^, regulators which foster the immune microenvironment in cancer^5^. With this in mind, it is not surprising that markers of immune escape are often associated with tumor infiltrating lymphocytes and mismatch repair (MMR) deficiency^6^.

VISTA, a member of the B7 family, was initially believed to be a negative immune checkpoint^7^. As of now, some studies on melanoma^8,9^ have described the suppressive effects of VISTA, with a presumed efficacy for anti-VISTA therapy. However, VISTA is highly controversial; it acts as a ligand on antigen-presenting cells, while serving as a receptor on T cells^10^. Many mouse models have shown that VISTA is highly expressed on naïve T cells, here a loss of VISTA expression impacts immune tolerance, further impacting cancer growth^7^. Evaluating VISTA in the setting of the tumoral microenvironment may be of value.

Recent work on the tumor invasive front includes both tumor budding (TB)^11^ and stromal differentiation (SD)^12^. High TB has been shown to be associated with worse prognosis outcomes in CRC^11^, although some believe that high TB may a consequence of immature SD, which could better represent the metastatic and mesenchymal CRC phenotype^13^. Histologically, the clinical significance of SD has been demonstrated in the breast^14^, cervix^15^ and esophagus^16^; however, it has been most extensively studied in cancers of the colon and rectum^17–20^.

QuPath is an open-source application for digital pathology and WSI analysis^21^. QuPath offers two major methods for biomarker analysis: superpixel image segmentation (SIS) and automatic cell count. In our study, we will utilize SIS for VISTA evaluation. For quality control, we will compare and correlate SIS with manual analysis.

This study is the first to use digital image analysis (DIA) to evaluate VISTA expression in CRC and it is also the first to analyze SD in relation to VISTA expression. Today, the relationship of VISTA expression to the tumoral microenvironment remains largely speculative. Understanding the role of VISTA in CRC is important, especially considering that VISTA may function differently in different tumor subtypes.

## Materials and methods

### Institutional Review Board

This study received approval for all experimental protocols by the Institutional Review Board (IRB) of the Human Research Protection Program licensing committee at Northwell Health. All methods were carried out in accordance with all guidelines and regulations. Informed consent waived due to the retrospective nature of the study was approved by the institutional review board (IRB) committee (Northwell Health IRB number: 18-0128).

### Study design

This study was retrospective, and we selected only primary resection specimens performed in our health system. We aimed to avoid the potential of small sample size which can result in wide confidence intervals (CI) and risk of errors in statistical analyses. We aimed for a sample size of over 200 and selected a case selection interval of 37 months in order to facilitate this: November 2014–December 2017. We searched in the pathology database (Cerner Millennium) for resection specimens with keywords “colon adenocarcinoma”, “rectal adenocarcinoma”, “adenocarcinoma of colon”, and “adenocarcinoma of rectum”. Cases with completed synoptic summaries and documented staging information were selected from the database consecutively. Cases diagnosed with Tis stage were excluded because these cases were considered lacking representative desmoplastic stroma. Cases lacking clinical information, appropriate follow-up, or tissue specimen availability were also excluded. No other specific stratification or matching by stage of disease or age was employed. One representative block was selected per case from a single slide containing the largest portion of tumor. VISTA immunohistochemical (IHC) expression was evaluated on these blocks. Hematoxylin and Eosin (H&E) stained slides were also evaluated for stromal differentiation, tumor budding and tumor-infiltrating lymphocytes*.* Further clinicopathological data was collected from the electronic medical records and patient follow-up data was collected from the Northwell Cancer Registry Database by the cancer registry at Northwell Health.

The primary end point of this retrospective cohort analysis was to evaluate the role of VISTA on cancer-free survival (CFS), defined by the time to death, recurrence or second primary. The secondary end points of this study were to determine the relationship between VISTA expression and the pathological and clinical profile. In exploratory analyses VISTA expression was compared to multiple variables including cancer-free survival, age, gender, pre-chemotherapy condition, pre-cancer condition, AJCC pathologic TNM stage, tumor budding score, tumor-infiltrating lymphocytes (TIL), tumor grade, stromal differentiation, mismatch repair (MMR) status, Ki-*ras2* Kirsten rat sarcoma viral oncogene homolog (KRAS) and B-Raf Proto-Oncogene (BRAF) mutational status.

### Immunohistochemistry

All the staining was performed on formalin-fixed and paraffin-embedded tissue blocks using the Ventana Benchmark Autostainer and Optiview detection kits (Ventana Medical System, Tucson, Arizona) at the Immunopathology Laboratory of Long Island Jewish Medical Center (Northwell Health System, New Hyde Park, NY). The antibody assay for VISTA (Cell signaling, D1L2G) was used at 1:200 dilutions with an antigen retrieval time of 40 min and an antibody incubation time of 32 min. The positive control for VISTA was lymph node tissue and the negative control was normal colon.

The MMR clones came pre-diluted from the manufacturer. MLH-1 (Clone M1, #790-5091, U OptiView DAB IHC v6, protocol #751: primary antibody incubation time: 32 min; Hematoxylin: 4 min; Bluing reagent: 4 min). MSH2 (Clone G219-1129, #790-5093, U OptiView DAB IHC v6, protocol #755: primary antibody incubation time: 32 min; Hematoxylin: 4 min; Bluing reagent: 4 min). PMS2 (Clone A16-4, #790-5094, OptiView DAB IHC v6, protocol #755: primary antibody incubation time: 32 min; OV HQ UNIV LINKR: 8 min; OV HRP MULTIMER: 8 min; OV AMPLIFIER: 4 min; Hematoxylin: 4 min; Bluing reagent: 4 min). MSH6 (Clone SP93, #790-5094, U OptiView DAB IHC v6, protocol #757: primary antibody incubation time: 20 min; Hematoxylin: 4 min; Bluing reagent: 4 min). The positive control for the foregoing MMR antibody clones was colon and no negative control was required for MMR according to manufacturer protocols.

### Slide digitalization

Histological features in this study were assessed using virtual slides. Slides were scanned on the Leica Aperio AT2. Vendor: Leica Biosystems, Buffalo Grove, Illinois, USA. Whole slide images are scanned at 20 ×. The Aperio vendor agnostic whole slide image viewer was used by pathologists in our study.

### Manual analysis of VISTA expression

Manual analysis was performed using virtual slides and VISTA immunohistochemistry was scored within tumor cells and stromal cells for percentage tissue involvement as according to VISTA OptiView protocol. Cytoplasmic and or membranous unequivocal staining of intensity above background was considered positive. Negative staining was characterized by the absence of any detectable IHC staining, characterized by a pale grey discoloration in tumor and stromal components.

### Superpixel image segmentation of VISTA expression

QuPath^21^ is an open source for whole slides image analysis which fosters multifaceted applications for image analysis in pathology. We utilized the superpixel method (SIS) available on QuPath version 0.2 and 20 × hotspots were identified from the whole slides image (WSI) by a surgical pathologist.

**Figure 1:**
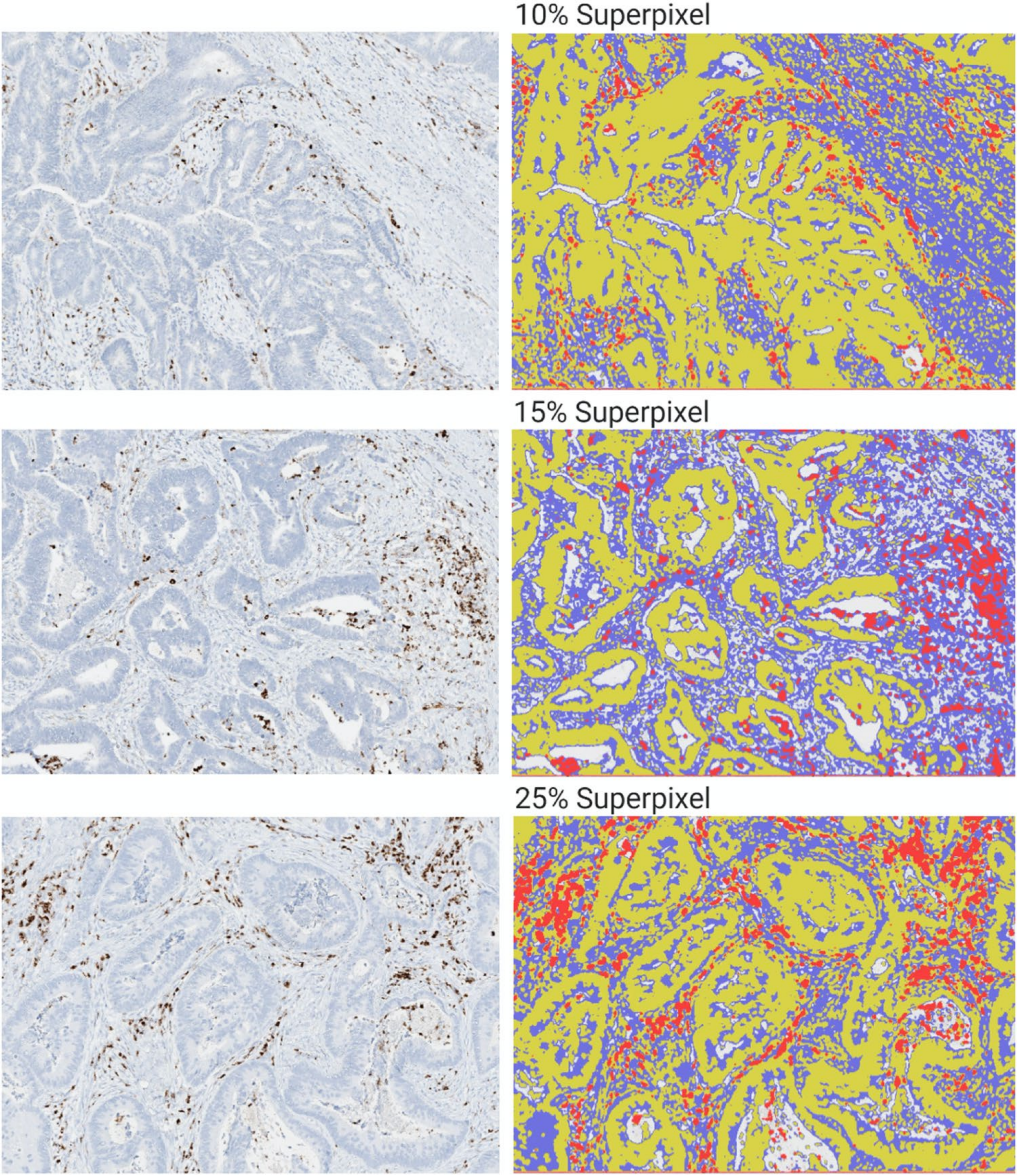
Superpixel analysis of VISTA expression. Red, VISTA; Yellow, Tumor; Blue. VISTA expression and immature stromal differentiation. *VISTA* V-domain immunoglobulin suppressor of T cell activation.

The superpixel method groups pixel similarity between different cellular populations^13,22^. This required manual annotation of each hotspot for training of the machine learning classifier to classify the superpixels accordingly. Components were selectively labeled and categorized as tumor (yellow), stroma (blue) and VISTA (red) through the generation of superpixel heatmaps. Quality control (QC) was performed consistently and manually by comparing the generated heatmap with the IHC hotspot images allowing for optimal classification of each VISTA hotspot. Once the ideal heatmap was generated, the classifier data could be saved in system for future use. Figure 1 demonstrates varying degrees of VISTA expression by SIS.

### AJCC staging

The primary tumor stage was staged as per AJCC 7th edition protocol as follows: pTis (Carcinoma in situ: intraepithelial or invasion of lamina propria); pT1 (Tumor invades the submucosa); pT2 (Tumor invades the muscularis propria); pT3 (Tumor invades through the muscularis propria into pericolorectal tissues); pT4a (Tumor penetrates to the surface of the visceral peritoneum); pT4b (Tumor directly invades or is adherent to other organs or structures). Lymph node status was staged as follows: pN0 (No regional lymph node metastasis); pN1a (Metastasis in one regional lymph node) pN1b (Metastasis to two to three regional lymph nodes); pN1c (Tumor deposits in the subserosa, mesentery, or non-peritonealized pericolic or perirectal tissues without regional nodal metastasis); pN2a (Metastasis in four to six regional lymph nodes); pN2b (Metastasis to 7 or more regional lymph nodes). pM1a (Metastasis confined to one organ); pM1b (Metastases in than one organ/sites or peritoneal metastasis is identified). Overall disease stages were classified based on AJCC 7th edition using the following criteria: Stage I (pT1, N0, M0) or (pT2, N0, M0); IIA (pT3, N0, M0); IIB (pT4a, N0, M0); IIC (pT4b, N0, M0); IIIA (pT1–T2, N1/N1c M0) or (pT1, N2a, M0); IIIB (pT3–T4a, N1/N1c, M0) or (pT2–T3, N2a, M0) or (pT1–T2, N2b, M0); IIIC (pT4a, N2a, M0) or (pT3–T4a, N2b, M0) or (pT4b, N1–N2, M0); IVA (Any pT, any pN, M1a); IVB (Any pT, any pN, M1b).

### Tumor budding

The assessment of tumor budding was based on the International Tumor Budding Consensus Conference (ITBCC) recommendations^23^. More specifically, a detailed search was done for the area having the highest grade of tumour budding. The counting of the buds was performed under 20 × objective lens hotspot region. According to ITBCC protocol, the tumor budding was graded into 3-tiers: Bd1: 0–4 buds, Bd2: 5–9 buds and Bd3: 10 or more buds.

### Tumor-infiltrating lymphocytes

TILs were defined as small blue mononuclear cells which infiltrating between tumor cells. Tumors were assessed with a 4-tier scale at the deepest point of the invasive tumor. This was previously validated for the quantification of inflammatory in colorectal cancer by Klintrup et al.^24^. A score of 0 denoted nil inflammatory cells, 1 denoted mild patchy increase in mononuclear cells, while 2 and denoted a moderate (bandlike) and 3 a florid (cuplike) inflammatory infiltrate, respectively. Scores 2 and 3 frequently are accompanied by destruction of cancer cell islands. Scoring was classified as low grade (0–1) and high grade (2–3).

### Stromal differentiation

For stromal differentiation, scoring was based on the grading system proposed by Ueno et al.^9^. We analyzed the extramural desmoplastic front at low magnification (4 ×). As according to Ueno protocol^9^ myxoid stroma was defined as an amorphous stromal substance made of amphophilic material with a basophilic to grey extracellular matrix and intermixed with randomly oriented hyalinized collagen. As in Ueno et al. stroma grading system, stroma was regarded as immature when fibrotic stroma with myxoid changes (> 40 × field) was observed. We categorized stroma as mature when the fibrotic stroma did not contain significant myxoid degeneration (< 40 ×), most comprised of fine mature collagen fibers stratified into multiple layers.

### Mis match repair status

MMR status was determined based of manual analysis of immunohistochemical protein expression. As according to the OptiView protocol, cases showing less than 1% of carcinoma nuclei immunohistochemical staining for any of the following stains: MLH1, PMS2, MSH2 and MSH6 were considered MMR deficient. Staining percentage was scored within tumor cells compared with tissue. Positive staining was classified as tumor cells exhibiting unequivocal nuclear staining above background. While the absence of any detectable signal, tan discoloration, pale grey in tissue sections was classified as negative.

### Next generation genomic sequencing

Molecular testing was performed on a subset of patients: 30 (13%) underwent BRAF testing and 34 (15%) underwent molecular testing for KRAS. Genomic alterations of BRAF and KRAS were tested by next generation genomic sequencing on formalin-fixed, paraffin embedded tissue. Mutational analysis was performed at Genpath laboratories (Elwood Park, NJ). Nucleic acid from the submitted specimen with a non-degraded or amplifiable concentration greater than 1 ng/μL was subjected to PCR-based amplification. Coding and non-coding regions of the selected genes were enriched and subsequently sequenced on an Illumina MiSeq instrument (San Diego, CA) with paired end, 175 base pair reads. Following mapping of the read data to the human genome (reference build GRCh37/hg19), single nucleotide variants, insertions and deletions with an allele frequency greater than 5% were detected utilizing a customized bioinformatics analytical pipeline.

### Statistical analysis

Pearson’s correlation coefficient was utilized for correlation between manual and superpixel analysis. Non-linear regression of cancer-free days and VISTA expression was used to optimize a cut-off value for VISTA expression. Comparative analysis was performed using the non-paired *t* test to examine the means of VISTA expression. When there were more than 2 groups in the category, *t* test was used to compare between each two groups. For pre-surgery therapy condition, we compared no chemotherapy group with partial regression group, no chemotherapy group with no regression group and partial regression group with no regression group. For pre-cancer condition, we compared non-adenoma group with tubular adenoma group, non-adenoma group with tubulovillous adenoma group, non-adenoma group with sessile serrated adenoma group, tubular adenoma group with tubulovillous adenoma group, tubular adenoma group with sessile serrated adenoma group and tubulovillous adenoma group with sessile serrated adenoma group. For pathological stage, T test was used to compare pT1 group with pT2 group, pT1 group with pT3 group, pT1 group with pT4 group, pT2 group with pT3 group, pT2 with pT4 group and pT3 with pT4 group. T test was also used to compare MMR intact group with MLH1/PMS2 mutation group, with MSH2/MSH6 mutation group and MLH1/PMS2 mutation group with MSH2/MSH6 group. Comparisons between VISTA subgroups and their clinicopathologic profile were performed using the Fisher’s exact tests. The Kaplan–Meier method was used to evaluate the VISTA expression and cancer-free survival rate as a function of time. The log-rank method was used to compare differences between the survival groups. The cox-regression univariate and multivariate analyses were utilized to calculate the predictors of survival, in which hazard rations (HRs) and confidence intervals (CIs) were analyzed. Statistical Analysis was performed using IBM SPSS 1.0.0.1508 and graphs were made on Prism Graphpad version 8.4.2 A P-value < 0.05 was considered statistically significant.

## Results

### Clinicopathologic and patient characteristics

A total of 231 cases of colorectal carcinoma were retrospectively analyzed and five cases were excluded due to inadequate tissue availability. The final study cohort comprised data from 226 patients with colorectal adenocarcinoma who underwent surgical resection at our health system. Surgeries included block resection, right hemicolectomy, left hemicolectomy, transverse colectomy, sigmoidoscopy, rectosigmoidectomy and abdominal perineal resection. The mean age for our patient cohort was 67.4. There was a slight male predominance: 49% female (108) to 51% male (118). 13 patients had neoadjuvant chemotherapy. 6 had partial regression whereas 7 were resistant to chemotherapy based on pathology reports. 138 patients had adenocarcinoma arising without an apparent associated polyp while 88 adenocarcinoma cases arose from polyps including tubular adenoma (65), villous adenoma (1), tubulovillous adenoma (20), and sessile serrated lesion (2). Disease stage were graded as following: I/II (123), III/IV (103).Tumoral stage was as follows: pT1 (26), pT2 (33), pT3 (110), pT4 (57). For TIL, scoring was as follows: Low (159) and High (67). Lymph node status was as follows: N0 (140), N1 (53), N2 (33). Tumor grade was as follows: Well-differentiated (161), Poorly differentiated (65). Tumor budding grades were as follows: Absent or low (113). Intermediate or high (113). Lymphovascular invasion status were as following: Not present (127), present (99). Stromal differentiation was as follows: 115 (50.9%) mature stroma, and 111 (49.1%) patients with immature stroma. Out of 226 patients, 179 had intact mismatch repair, while 47 patients were mis match repair deficient. Molecular testing was performed on a subset of patients: 30 (13%) had BRAF testing and 34 (15%) had KRAS mutation status.

### VISTA expression and positivity cut-off

**Figure 2:**
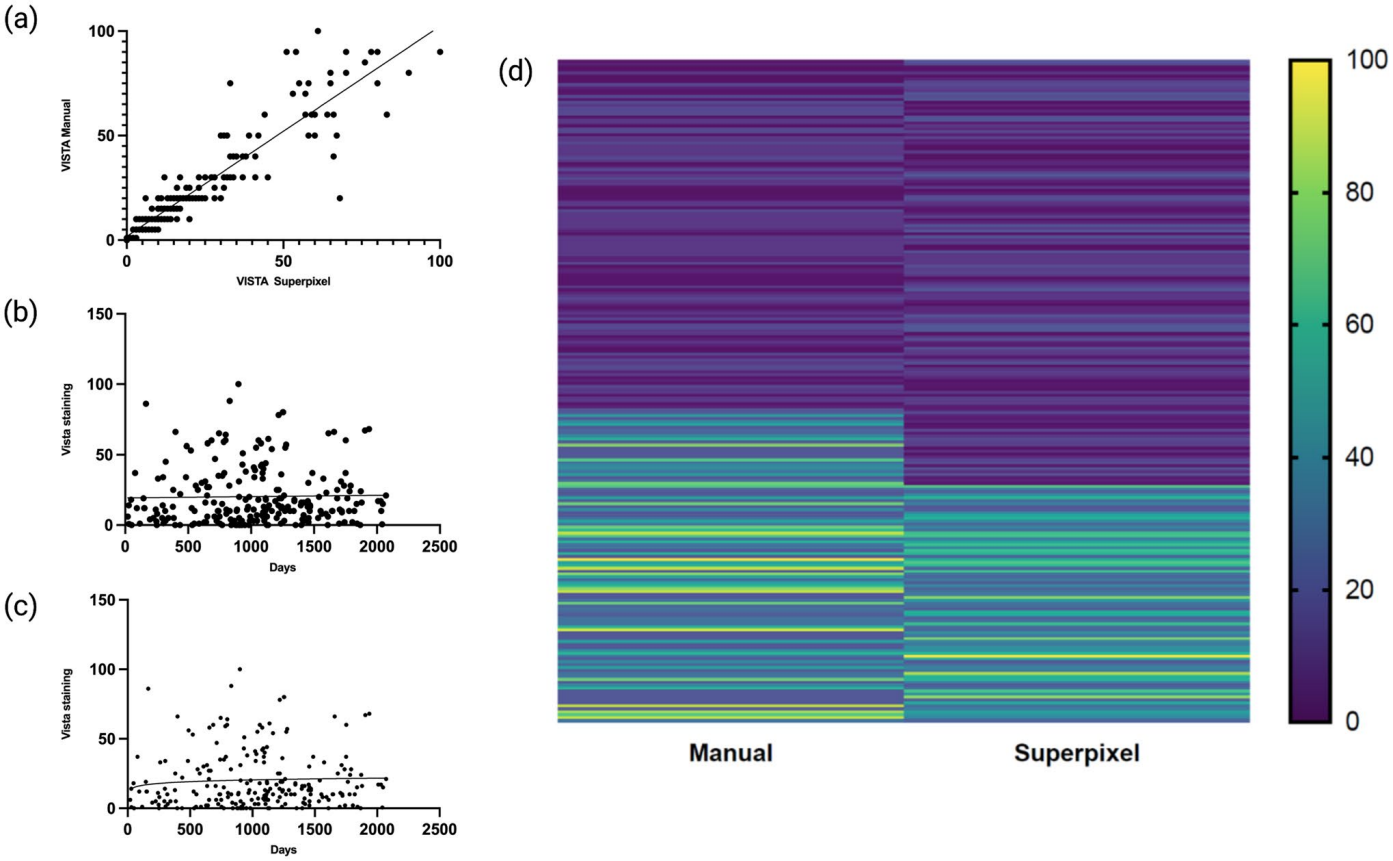
(**a**) Pearson’s correlation between manual and superpixel analysis of VISTA expression. (**b**) Nonlinear regression for VISTA expression and CFS by manual analysis. (**c**) Nonlinear regression for VISTA expression and CFS by superpixel analysis. (**d**) Heatmap for VISTA expression for manual and superpixel analysis.

The overall mean expression of VISTA on superpixel analysis was 20.3%, while 20 cases showed 0% expression and 5 cases showing more than 80% expression. The overall expression of VISTA on manual analysis was 22.8%, 16 cases showed 0% expression and 10 cases showing more than 80% expression. Pearson’s correlation coefficient demonstrated good correlation between manual and superpixel analysis (r = 0.92), shown in Fig. 2a. Nonlinear regression found the optimal cutoff for VISTA staining and survival to hover at 20.3% expression for both manual as seen in Fig. 2b, and for superpixel analysis as seen in Fig. 2c. Heatmaps for VISTA expression between manual and superpixel analysis can be seen in Fig. 2d. Positive expression was classified as greater than 20% for both manual and superpixel analysis. For manual analysis, the total number of positive VISTA expression cases was 70; for superpixel image segmentation, a total of 75 cases were classified as positive expression.

### VISTA expression and variables

The following factors were selected to compare the mean VISTA expression: Age, gender, pre-surgery status, pre-cancer condition, disease stage, pathologic T stage, lymph node stage, tumor grade, tumor budding, LVI, TILs, and stroma differentiations. *t* test was conducted to compare the mean VISTA expression in each group. For pre-surgery therapy, partial regression group was found to be associated with high VISTA expression when compared to the no regression group on manual (P = 0.03) and superpixel analysis (P = 0.02). High AJCC stage (III/IV) was found to associated with low mean VISTA expression on both manual (P = 0.0249) and superpixel analysis (P = 0.0386). For pathologic tumoral stage, pT1 was found to have the highest VISTA expression, and was significantly higher than pT2 (P = 0.004 on manual and P = 0.05 on superpixel), higher than pT3 (P = 0.003 on manual and P = 0.046 on superpixel) and pT4 (P = 0.001 on manual and P = 0.025 on superpixel) on *t* test. Whereas for mean VISTA expression among pT2 and pT3 as well as pT3 and pT4, there was no significant difference. High tumor grade was associated with low VISTA expression on manual analysis (P = 0.049) but not on superpixel analysis. High TIL scoring was found to correlate with higher mean VISTA expression on both manual analysis (P = 0.049) and superpixel analysis (P = 0.037). When comparing stroma differentiation groups, mature stroma was associated with high VISTA expression both by manual (P = 0.0041) and superpixel analysis (P = 0.00091). Age, gender, lymph node status, and tumor budding groups did not have significant VISTA expression differences intergroup (P > 0.05). For biomarker status, we found that BRAF mutation group was more likely to have a high mean VISTA expression, with superpixel analysis showing a significant difference (P = 0.05). KRAS mutation status were not found to be associated with VISTA expression (P > 0.05). When divided the MMR status into MLH1/PMS2 loss group and MSH2/MSH6 loss group, MLH1/PMS2 loss group was associated with higher VISTA expression both on manual (P = 0.001) and superpixel analysis (P = 0.001) when compared to the MMR intact group; MSH2/MSH6 loss group was also found to be associated with high VISTA expression both on manual (P = 0.001) and superpixel analysis (P = 0.001) when compared to the MMR intact group; VISTA expression among MLH1/PMS2 loss and MSH2/MSH6 loss groups were not found to be associated with VISTA expression (P > 0.05). Detailed VISTA mean staining was analyzed by unpaired *t* test and the results were shown in Table 1.

**Table 1:** Unpaired *t* test for mean vista expression. Significant features (P ≤ 0.05) are shown in bold.

Variable	Frequency	Manual	P-value				Superpixel	P-value			
VISTA expression		Mean					Mean				
**Age**			0.0853					0.331			
≤ 70	132	20.58					19.18				
> 70	94	26.05					21.91				
**Gender**			0.063					0.093			
Male	118	25.37					22.77				
Female	108	20.10					17.63				
**Pre-chemotherapy**											
No therapy	213	22.89	No therapy vs		PR vs NR		20.28	No therapy vs		PR vs NR	
Complete regression	0	–					–				
Partial regression	6	23.36	0.175		**0.03**		27.01	0.156		**0.02**	
No regression	7	12.50	0.162				15.5	0.145			
**Pre-cancer condition**											
Non	138	22.40	Non vs	TA vs		TVA vs	19.792	Non vs	TA vs		TVA vs
TA	65	22.38	0.646				19.526	0.672			
VA	1	20	–	–		–	20	–	–		–
TVA	20	25.45	0.232	0.102		0.301	27.140	0.06	0.054		0.057
SSA	2	45.00	0.507	0.402			15.552	0.267	0.231		
**AJCC stage**			**0.0249**					**0.0386**			
I/II	123	25.70					21.05				
III/IV	103	20.84					19.74				
**Pathologic stage**			pT1 vs	pT2 vs		pT3 vs		pT1 vs	pT2 vs		pT3 vs
PT1	26	37.19					28.96				
PT2	33	22.45	**0.004**				22.04	**0.05**			
PT3	110	22.97	**0.003**	0.535			20.02	**0.046**	0.410		
PT4	57	17.59	**0.001**	0.171		0.304	15.95	**0.025**	0.156		0.344
**Lymph node stage**			0.3147					0.745			
N0	140	24.09					20.67				
N1-2	86	20.84					19.74				
**Tumor grade**			**0.049**					0.074			
G1–G2	161	26.26					22.72				
G3	65	21.28					19.25				
**Tumor budding**			0.7550					0.1282			
TBD1	113	23.35					22.61				
TBD2 or TBD3	113	22.36					18.39				
**LVI**			0.696					0.472			
Not present	127	22.39					19.66				
Present	99	23.45					21.15				
**TIL**			**0.049**					**0.037**			
Low	159	20.85					18.45				
High	67	27.61					24.73				
**Stromal differentiation**			**0.0041**					**0.00091**			
Immature	111	18.30					16.66				
Mature	115	27.25					23.84				
**MMR status**											
Intact	187	19.77	Intact vs MSI		MSI groups compare		17.81	Intact vs MSI		MSI groups compare	
MLH1/PMS2 mutation	30	37.62	**0.001**				32.51	**0.001**			
MSH2/MSH6 mutation	9	55.67	**0.001**		0.3		49.67	**0.001**		0.14	
**KRAS**			0.195					0.405			
Wild type	19	26.21					21.16				
Mutation	15	15.13					15.43				
**BRAF**			0.138					**0.05**			
Wild type	16	12.60					10.287				
Mutation	16	22.86					23.286				

*VISTA* V-domain immunoglobulin suppressor of T cell activation, *TA* tubular adenoma, *TVA* tubulovillous adenoma, *VA* villous adenoma, *SSA* sessile serrated adenoma, *TBD* tumor budding grade, *LVI* lymph-vascular invasion, *PT* stage, *N* nodal stage, *TIL* tumor-infiltrating lymphocytes, *G* grade, *t* test for selected biomarkers, *MMR* mismatch repair gene, *KRAS* Ki-ras2 Kirsten rat sarcoma viral oncogene homolog BRAF, B-Raf Proto-Oncogene. Standard deviation (SD) and standard error (SE) for each group as follows in format factor (SD, SE): age: manual: ≤ 70 (22.212, 1.933), > 70 (25.156, 2.595), superpixel: ≤ 70 (19.572, 1.7); > 70 (22.39, 2.3); Gender: manual: male (23.85, 2.32) female (21.82, 2.1), superpixel: male (21.44, 2.08) female (19.15, 1.84); Pre-surgery treatment: manual: NA (23.69, 1.62), PR (24.83, 10.13), CR (8.60, 4.33); superpixel: NA (20.89, 1.43), PR (28.12, 11.48), CR (9.94, 4.97); Precancer condition: manual: Non (20.33, 1.73), TVA (31.04, 6.94), SSA (7.78, 5.5); Superpixel: Non (23.57, 2.06), TVA (28.67, 6.41), SSA (35.35, 25.0). AJCC stage: manual: I/II (22.27, 4.45), III/IV (27.91, 3.32), superpixel: I/II (22.59, 4.51), III/IV (23.51, 2.79). Tumor stage: manual: pT1 (27.521, 3.583), pT2 (21.502, 3743), pT3 (21.722, 1.681), pT4 (19.633, 2.601); superpixel: pT1 (26.825, 5.26), pT2 (23.5110, 3.0609), pT3 (19.8313, 1.8908), pT4 (19.549, 1.5127); LN: manual: N0 (24.17, 2.43) N1/2 (19.86, 2.67); superpixel: N0 (20.15, 1.7) N1/2 (20.66, 2.78). Tumor budding: manual: BD1 (24.03, 2.26), BD2–3 (23.22, 2.19); TIL: manual: low (23.196, 1.840), high (23.985, 2.93); superpixel: low (20.17, 1.599), high (21.7078, 2.652); Stromal maturation: manual: immature (21.486, 2.039), mature(24.749, 2.308), superpixel: immature (19.6581, 1.87), mature (21.3203, 1.9881); BRAF: manual: loss (19.848, 5.305), intact (28.819, 7.205), superpixel: loss (19.7951, 5.29), intact (19.2428, 4.8107); MMR: manual: intact (20.481, 1.527), loss (30.067, 4.482), intact (18.313, 1.365), loss (25.943, 3.867).

When dividing the VISTA expression into negative (≤ 20%) and positive (> 20%) expression group, positive VISTA expression group was associated with low AJCC stage (I/II) on both manual (P = 0.022) and superpixel (P = 0.001) analysis. Positive VISTA expression also was associated with high TIL infiltrates on manual (P = 0.001) and superpixel (P = 0.001) analysis as well as mature stromal differentiation (manual analysis P = 0.007 and superpixel P = 0.001). Positive VISTA expression did not have significant associations with other clinicopathologic features including age, gender, pre-surgery treatments, pre-cancer condition, lymph node stage, tumor grade, tumor budding, and LVI. For molecular status, VISTA expression happened more often in MMR loss status (manual analysis P = 0.003, superpixel P = 0.003) and BRAF mutated status (manual analysis P = 0.001, superpixel P = 0.031). Detailed Fisher-exact results are shown in Table 2.

**Table 2:** Unpaired *t* test for mean vista expression. Significant features (P ≤ 0.05) are shown in bold.

Variable	VISTA manual negative	VISTA manual positive	P-value	VISTA superpixel negative	VISTA superpixel positive	P-value
**VISTA expression**						
**Age**			0.086			0.817
≤ 70	97	35		89	43	
> 70	59	35		62	32	
**Gender**			0.100			0.167
Male	75	43		73	45	
Female	81	27		78	30	
**Pre-surgery chemotherapy**			0.880			0.899
No therapy	146	67		142	71	
Complete regression	0	0		0	0	
Partial regression	7	2		6	3	
No regression	3	1		3	1	
**Pre-cancer condition**			0.918			0.938
Non	94	44		92	46	
TA	1	1		1	1	
VA	46	19		44	21	
TVA	14	6		13	7	
SSA	1	0		1	0	
**AJCC stage**			**0.022**			**0.001**
I/II	71	42		67	46	
III/IV	85	28		84	29	
**Pathologic stage**			0.099			0.052
PT1	12	5		13	4	
PT2	14	4		15	3	
PT3	83	48		79	52	
PT4	47	13		44	16	
**Lymph node stage**			0.719			0.753
N0	96	44		92	48	
N1–2	60	26		59	27	
**Tumor grade**			0.232			0.292
G1–G2	115	45		111	49	
G3	41	25		40	26	
**Tumor budding**			0.670			0.757
TBD1	92	44		87	49	
TBD2 or TBD3	64	26		64	26	
**LVI**			0.498			0.541
Not present	90	37		87	40	
Present	66	33		64	35	
**TIL**			**0.001**			**0.001**
Low	97	15		96	16	
High	59	55		55	59	
**Stromal differentiation**			**0.007**			**0.001**
Immature	86	25		87	24	
Mature	70	45		64	51	
**MMR status**			**0.003**			**0.003**
Intact	137	50		133	54	
Loss	19	20		18	21	
**KRAS**			0.867			0.667
Wild type	14	5		14	5	
Mutation	11	4		11	4	
**BRAF**			**0.001**			**0.031**
Wild type	15	1		14	2	
Mutation	5	11		7	9	

*VISTA* V-domain immunoglobulin suppressor of T cell activation, *TBD* tumor budding grade, *PT* stage, *N* nodal stage, *LVI* lymph-vascular invasion, *TIL* tumor-infiltrating lymphocytes, *G* grade, *t* test for selected biomarkers, *MMR* mismatch repair gene, *KRAS* Ki-ras2 Kirsten rat sarcoma viral oncogene homolog, *BRAF* B-Raf Proto-Oncogene.

### VISTA expression and cancer-free survival

**Figure 3:**
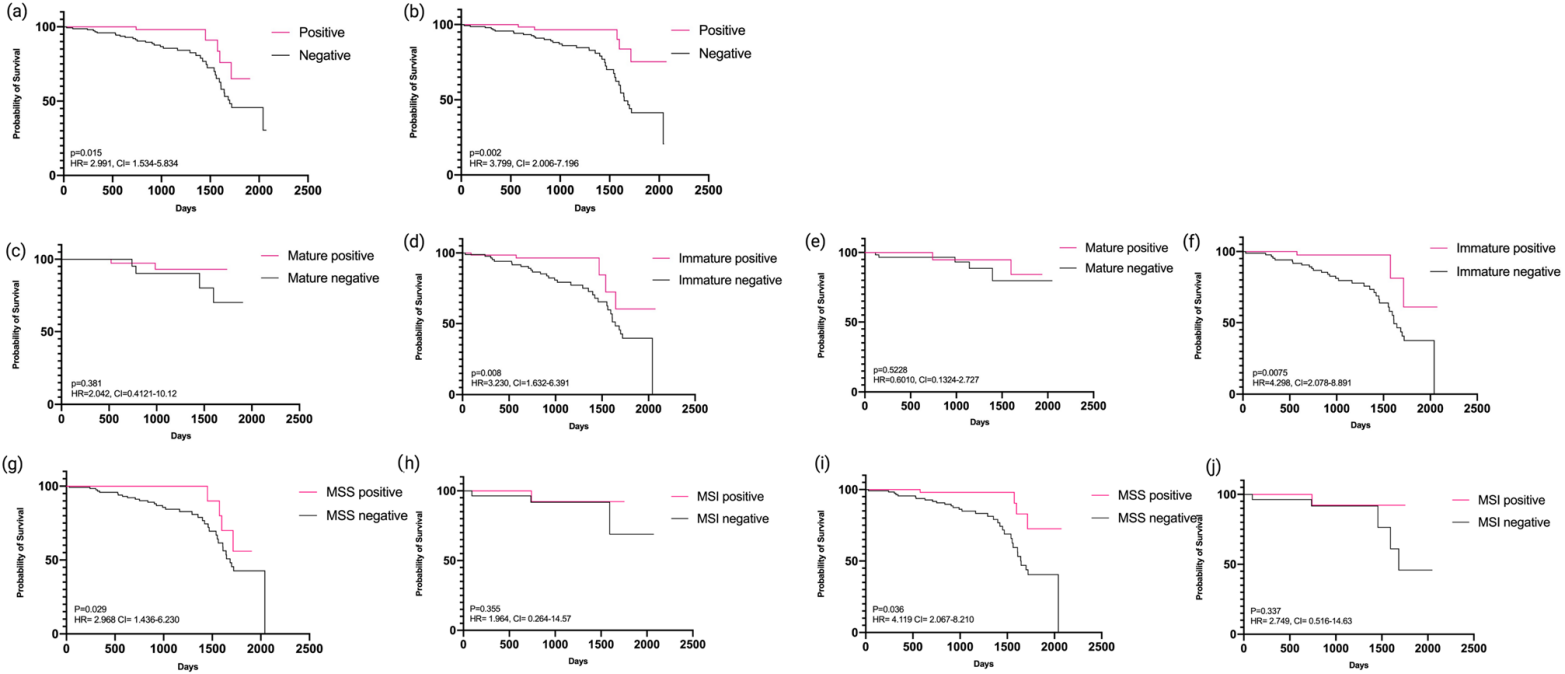
Kaplan–Meier survival for VISTA expression by manual and superpixel analysis with P values, Hazard Ratio and Confidence in shown in the figures. (**a**) Total survival rate between VISTA positive and negative group with manual analysis. (**b**) Total survival rate between VISTA positive and negative group with superpixel analysis. (**c**) Manual analysis of VISTA expression and survival analysis in mature stroma. (**d**) Manual analysis of VISTA expression and survival analysis in immature stroma. (**e**) Superpixel analysis of VISTA expression and survival analysis in mature stroma. (**f**) Superpixel analysis of VISTA expression and survival analysis in immature stroma. (**g**) Manual analysis of VISTA expression and survival analysis in microsatellite stability. (**h**) Manual analysis of VISTA expression and survival analysis in microsatellite instability. (**i**) Superpixel analysis of VISTA expression and survival analysis in microsatellite stability. (**j**) Superpixel analysis of VISTA expression and survival analysis in microsatellite instability.

Cancer free survival (CFS) data was collected for all of the 226 patients with a mean follow up time of 1054 days. After setting the positivity cutoff to greater than 20%, we found that positive VISTA expression to be associated with favorable CFS by manual analysis (P < 0.015) and superpixel image segmentation (P = 0.002), as shown in Fig. 3. For manual analysis, the mean cancer-free period for VISTA expression > 20% was 1648 days (95% confidence interval: 1689–1875 days), 134 days longer than those with VISTA expression <  = 20% (95% confidence interval: 1543–1753 days). During the follow-up period, 92.9% cases with positive VISTA were cancer-free compared to the 76.3% cancer-free rate in patients negative for VISTA. For superpixel image segmentation, positive VISTA expression was associated with a mean CFS of 1932 days (95% confidence interval: 1820–2043 days), 312 days longer than those with negative VISTA expression. During the follow-up period, 93.3% cases were positive for VISTA, compared to the 75.7% CFS for those with negative VISTA expression.

When CFS and VISTA expression was separated based on SD, VISTA was shown to not be significant in patients with mature SD (P > 0.05). However, positive VISTA expression was found to be associated with CFS in patients with immature SD on both manual (P = 0.008) and superpixel analysis (P = 0.007).

When analyzing based on MMR status, positive VISTA was found to be associated with longer CFS in MMR intact patients on both manual (P = 0.029) and superpixel analysis (P = 0.036). However, VISTA was found not to be associated with CFS differences in MMR loss status (P > 0.05). Kaplan–Meier survival analyses for VISTA expression and SD can be viewed in Fig. 3.

Based upon cox-regression of cancer-free survival (CFS), positive VISTA expression in superpixel analysis was found to associated with better prognostic outcomes on univariate (P = 0.005) and multivariate analyses (P = 0.006). Advanced disease stage (stage III/IV) was associated with worse prognosis on univariate analysis (P = 0.038) and on multivariate analysis (P = 0.001). High T stage was associated with poor CFS on both univariate analysis (P = 0.018) and multivariate analysis (P = 0.009). Tumor budding was found to be a poor prognostic factor on univariate analysis (P = 0.048) but not multivariate analysis (P = 0.108). Lymph vascular invasion was associated with poor prognosis on univariate analysis (P = 0.048), but not on multivariate analysis (P = 0.072). Meanwhile, SD was found to be significantly associated with CFS on both univariate (P = 0.008) and multivariate analysis (P = 0.003). The remaining clinicopathological variables were not significant (P > 0.05) on cox proportional hazard regression analysis (Table 3).

**Table 3:** Univariate and multivariate analyses of cancer free survival using the Cox proportional-hazard regression.

Variable	Univariant	CI	P-value	Multivariant	CI	P-value
**VISTA Manual analysis**	**0.331**	**0.130–0.845**	**0.021**	–	–	–
≤ 20%						
> 20%						
**VISTA Superpixel analysis**	**0.262**	**0.103–0.668**	**0.005**	**0.95**	**0.902–0.987**	**0.006**
≤ 20%						
> 20%						
**Age**	1.024	0.990–1.050	0.062	0.989	0.955–1.024	0.524
≤ 70						
> 70						
**Gender**	1.077	0.587–1.976	0.810	1.325	0.652–2.693	0.438
Male						
Female						
**Pre-surgery chemotherapy**	1.601	0.872–2.941	0.129	1.513	0.813–2.815	0.191
No therapy						
Complete regression						
Partial regression						
No regression						
**Pre-cancer condition**	0.985	0.866–1.119	0.814	1.014	0.890–1.157	0.830
Non						
TA						
VA						
TVA						
SSA						
**AJCC stage**	**2.114**	**1.312–10.62**	**0.038**	**35.235**	**8.926–39.089**	**0.001**
I/II						
III/IV						
**Pathologic stage**	**5.619**	**1.352–23.356**	**0.018**	**7.306**	**1.645–32.452**	**0.009**
PT1						
PT2						
PT3						
PT4						
**Lymph node stage**	1.091	0.698–1.704	0.702	1.156	0.687–1.945	0.586
N0						
N1-2						
**Tumor grade**	1.442	0.730–2.851	0.292	2.405	0.883–4.736	0.095
G1-G2						
G3						
**Tumor budding**	**2.180**	**1.638–3.281**	**0.048**	1.180	0.564–2.469	0.660
TBD1						
TBD2 or TBD3						
**LVI**	**1.946**	**1.007–3.763**	**0.048**	1.356	0.921–4.342	0.072
Not present						
Present						
**TIL**	1.010	0.533–1.911	0.977	2.045	0.883–4.736	0.095
Low						
High						
**Stromal differentiation**	**3.062**	**1.342–6.989**	**0.008**	**3.990**	**1.604–9.924**	**0.003**
Immature						
Mature						
**MMR (IHC)**	0.823	0.404–1.678	0.593	1.679	0.599–4.707	0.325
All positive						
Loss of MLH1/PMS2						
Loss of MLH1 only						
Loss of PMS 2 only						
Loss of MSH2/MSH6						
Loss of MSH2 only						
Loss of MSH6 only						

*HR* hazard ratio, *VISTA* V-domain immunoglobulin suppressor of T cell activation, *TBD* tumor budding grade, *PT* stage, *N* nodal stage, *LVI* lymph-vascular invasion, *TIL* tumor-infiltrating lymphocytes, *G* grade, *MMR* mis-match repair. Significant features (P ≤ 0.05) are shown in bold.

## Discussion

Immune checkpoint targets have been studied extensively in cancer^25^; however, VISTA is a relatively novel immune checkpoint and the significance is not fully understood. In the alimentary tract, positive VISTA expression has been found to be associated with improved prognostic outcomes in esophageal and gastric adenocarcinomas^26,27^. Most recently, a broad meta-analysis of 10 studies was reported by He et al.^28^, 7 of the 10 studies revealed high expression of VISTA to be associated with a favorable prognosis, which also included solid tumors of the ovary as well as mesothelioma.

The present study found VISTA expression to correlate with stromal differentiation, a novel concept which was initially proposed by Ueno et al.^17^. In recent years, several studies have described the stroma differentiation to be associated with prognostic outcomes in colonic adenocarcinoma^17^. In our study, we found that positive VISTA expression to be correlated with mature SD, and when cohorts were separated based on SD, only VISTA expression in patients with immature SD were found to correlate with CFS. Tumor stage and TILs were also found to correlated with VISTA expression, further suggesting VISTA to be associated with pathologic features in CRC. Our study suggests this phenotype to include high VISTA expression, lower tumor stage, mature SD, higher TILs and longer patient survival.

Regarding the findings in our study, we found high VISTA expression to predict positive outcomes in colon cancer. There are several hypotheses to explain this. Firstly, VISTA is found on myeloid-derived suppressor cells (MDSCs), CD4+ T cells, and FoxP3+ regulatory T cells (Treg)^29^. VISTA has been demonstrated by Lines, J. L. et al.^29^ for its function of converting naïve T cells into FoxP3+ T regulatory cells. Secondly, Sun et al.^30^ has shown FoxP3+ T cells as a protective factor in colon cancer. In a large cohort for chemotherapy patients, FoxP3+ T cell were found to be associated with favorable outcomes, and patients with high FoxP3+ infiltrates benefited more from chemotherapy^31^. Pagano et al.^32^ revealed FoxP3+ cells function as a transcriptional repressor of SKP2 and regulates the G2/M phase cell cycle. Inhibiting the iFoxP3+ T cells results in increased cell proliferation^30,32^. Thus, VISTA may function as a protective factor by increasing FoxP3+ T cell infiltrates in the tumor microenvironment.

VISTA has been reported as a protective factor in esophageal adenocarcinoma by Loeser et al.^26^. Muller et al.^40^ also reported VISTA in mesothelioma to be associated with better overall survival. However, VISTA expression in melanoma was found to be associated with a poor prognosis^8^, suggesting VISTA function may vary between different tumor types. Clinical trials will need to be carefully designed and will determine the therapeutic efficacy.

In our analysis of VISTA, QuPath derived superpixel analysis was used and compared with the standard manual analysis. Serving as an open access software, QuPath has been utilized in many studies^21^. It was adopted for deep learning based automatic detection of high-grade nuclei in cervical squamous intraepithelial neoplasia (CIN) by Sornapudi et al.^35^. QuPath contains two major methods in terms of cellular quantification: superpixel and automatic cell quantification. Superpixel method separates tissue components based on RBG values. Cell quantification recognizes expression based on cell shapes. However, superpixel analysis will provide more subcellular information, potentially helpful in analyzing stroma components. We compared the results with standard manual analysis and found superpixel analysis provided a more stable quantification and separated cohorts better.

Superpixel analysis has also been used in other fields of medical research. Huang et al.^33^ reported using superpixels to analyze breast ultrasound; and Tamajka et al. utilized superpixels on MRI images for assessing brain vascularitity^34^. In the future, superpixel based approaches could be validated and integrated into the daily clinical workflows for biomarker analysis.

Hotspot based analysis was reported by Robertsona et al.^36^ as an analyzing method adopted for whole slide images. Hotspots were found to correlate better with clinicopathological features and outperformed manual scoring in predicting survivals. Looking forward, hotspots method could be utilized and combined with future applications in deep learning.

There are several pitfalls in our study. Firstly, there was a possibility for selection bias due to the retrospective nature of our study. Secondly, we did not perform multiplex immune staining. This would have been helpful in determining a correlation of VISTA with CD4+ and FoxP3+ T cells. Finally, we were not able to validate our VISTA findings in a second cohort of CRC patients.

However, a recent publication by Zong et al.^39^ evaluated VISTA expression in tissue microarrays from a larger cohort of 1434 patients with stage I–III CRC. They also found that high VISTA expression correlated with low tumor stage, MMR deficiency, and favorable prognostic outcomes in patients with CRC. Our findings further validate VISTA as a clinically significant biomarker in CRC and also demonstrate the utility of SIS for biomarker analysis.

As for genetic profiling, we found that MMR status correlated with VISTA expression. Interestingly, once patients were subdivided based on MMR status, only VISTA expression in microsatellite stable patients was associated with longer CFS. There were no statistical differences observed in for microsatellite instability patients and CFS. This may be due to the fact that MSI tumors are more likely to have VISTA expression immune cells as shown in our study and MSI status generally represents a good outcome in colon cancer^37^. Numerous studies have shown MSI to have increase infiltrates of CD3+ and CD8+ T cells^37^, supporting the role of MMR as a complex regulator of the immune microenvironment, and the rational for ubiquitous analysis in CRC.

Our novel molecular finding was that BRAF mutations were associated with higher VISTA expression, although our BRAF cohort was small. However, one of the studies by Rosenbaum et al.^9^ revealed a possible explanation for this phenomenon. This study demonstrated VISTA expression to be negatively regulated by Forkhead box D3 (FOXD3), a downstream transcription factor in BRAF pathway. A finding which could explain the high VISTA expression seen in patients with BRAF mutational status. Although further studies between BRAF and VISTA need to be done, especially in the setting of melanoma, where VISTA expression could influence the efficacy of BRAF inhibitor therapy^38^.

For VISTA, there are many questions which remain. Future studies are needed to examine the therapeutic efficacy of VISTA based therapeutics, which could be used in combination with other immune check point inhibitors. Looking forward, targeting VISTA in an inhibitory fashion may be performed, but cautiously. It could be immune-protective for patients with colorectal cancer and its clinical significance may depend on the underlying tumoral microenvironment.
